# Strange bedfellows: bridging the worlds of academia, public health and the sex industry to improve sexual health outcomes

**DOI:** 10.1186/1478-4505-9-S1-S13

**Published:** 2011-06-16

**Authors:** Wendy Knerr, Anne Philpott

**Affiliations:** 1 Taking Action For Sexual Health, P.O. Box 1502, Oxford, OX4 9ED, UK

## Abstract

The public health response to sexually transmitted infections, particularly HIV, has been and continues to be overwhelmingly focused on risk, disease and negative outcomes of sex, while avoiding discussion of positive motivations for sex (e.g. pleasure, desire, love). Recent advocacy efforts have challenged this approach and organisations have promoted the eroticisation of safer sex, especially in the context of HIV prevention.

This paper is a case study of one of these organizations – The Pleasure Project. It gives a brief background on the public-health approach to sex and sexual health, and recommends an alternative approach which incorporates constructs of pleasure and desire into sexual health interventions. The Pleasure Project’s aims and unorthodox communications strategies are described, as are the response to and impact of its work, lessons learned and ongoing challenges to its approach.

The Pleasure Project combines evidence (rigorous and experimental as well as qualitative and anecdotal) with experiential knowledge from the sex industry and safer-sex promotion to communicate messages about eroticising safer sex to influence researchers, public health practitioners and policymakers, mainstream media and the porn world. There are significant barriers to this work, because it challenges common and entrenched norms and values related to sex and pleasure and their role in the public health sphere. Other barriers include: the limited range of existing rigorous intervention trials which incorporate pleasure constructs; the lack of effective indicators to measure pleasure constructs; limited funding and resources; discomfort among public health practitioners, researchers and donors with concepts of pleasure and sex; and rejection of erotic media as a potential tool for prevention.

Despite the backdrop of sex-negative public health practice, there is anecdotal evidence that safer sex, including condom use, can be eroticised and made pleasurable, based on qualitative research by The Pleasure Project and other like-minded organisations. Yet there is a need for more research on the effectiveness of pleasure components in sexual health interventions, particularly in high-risk contexts. This need has become urgent as practitioners look for new ways to promote sexual health and as new prevention technologies (including female condoms and microbicides) are introduced or disseminated.

## Background

The public health response to HIV has been and continues to be overwhelmingly focused on risk, disease and the negative outcomes of sex [1-3], while avoiding discussion of positive motivations for sex (e.g. pleasure, desire, love). This can be conceptualised as a ‘sex-negative’ approach, in that it treats human sexual behaviour as largely harmful or otherwise problematic, or as potentially having a negative influence on individuals and society. (This is in contrast to a ‘sex-positive’ approach, which sees sexual behaviour as inherently healthy and natural, and/or potentially beneficial, fun or having a positive influence on individuals and societies.)

Advocacy to challenge the sex-negative approach has come largely from proponents of comprehensive sexuality education, and more recently from organisations promoting the eroticisation of safer sex or the incorporation of constructs of sexual pleasure in interventions, especially in the context of HIV prevention. Literature suggests that pleasure (whether in relation to sex, drugs or other issues) has been under-examined in public health research and policy [4]. This has been linked, for example, to

“medical science’s preference for studying the causes of illness rather than health. It may also reflect more deep-seated difficulties with modern western ideas about pleasure and what is considered ‘serious’ scientific pursuit.” [[4], p162].

This paper is a case study of one organization – The Pleasure Project – which is involved in countering sex-negative approaches and bringing pleasure and eroticism into sexual health programming. The paper gives a brief background on the public-health approach to sex and sexual health, and outlines some of the barriers faced when challenging deep-set and commonly held norms around these issues. It then explains how The Pleasure Project communicates messages about eroticising safer sex to influence diverse audiences – from researchers to public health practitioners, and from the mainstream media to the porn world Finally, the paper assesses what has worked well, and less well, in efforts to put the ‘sexy’ into safer sex and sexual health promotion.

### Public-health approach to sexual health

As public health advocates and practitioners work to ‘sell’ the idea of good health, for example by promoting condom use, they compete with a much better funded, globalised media machine that sells everything – from cars to toothpaste, as well as sex and pleasure – using sex itself. If and when the pursuit of sexual pleasure is mentioned in health literature, it is characterised largely as destructive [5] or as a major contributor to the spread of disease, and therefore something to be controlled or suppressed [e.g., [6-8]].

In some cases, the AIDS pandemic has created an opportunity for more open discussion of sexuality, sexual behaviour, safer sex and other ordinarily taboo issues in many settings [e.g., [9,10]]. However, public health discourse and campaigns still tend to ignore sexual pleasure and desire, or to focus largely on negative aspects of sex and sexuality [11,12]. (It should be noted, however, that this phenomenon is not exclusive to public health – many cultures generally consider sex to be a destructive or dangerous force, and religious institutions often characterize sex as negative.)

Pleasure and eroticisation have been elements of grass roots-level HIV interventions at specific times and in particular contexts, for example, at the beginning of the HIV epidemic among men who had sex with men in some high-income countries, where eroticising safer sex was a key component of prevention programmes [13]. However, this has rarely been the case among other risk groups, in large-scale or state-sponsored programmes, or in the context of international development, where sex has been treated as something to be controlled and contained [11], and primarily in relation to population control, disease and violence [14,15].

Negative messages about sexuality can undermine, rather than promote, safer sexual behaviour. For example, a study from India showed that society’s condemnation of masturbation – fuelled by the belief that men are weakened when they ‘waste’ semen by ejaculating outside a woman’s body – prompted young men to satisfy their sexual desires by visiting sex workers or having male-to-male penetrative sex. This put them at risk for sexually transmitted infections (STIs), including HIV [[16], as referenced in Ingham [1]. Research about condom use, especially among heterosexual married couples, tends to highlight the association of condoms with illness or death, links with stigmatised behaviours such as ‘casual sex’ and commercial sex work, and inevitably reduces enthusiasm to use them [17]. The negative focus also limits, or indeed silences, the examination of alternative means of giving and receiving pleasure outside of heterosexual penetrative sex, such as mutual masturbation [1], which can be a much safer option.

Gender stereotypes also play a role in demonising sex and pleasure in public health, by positioning men as predators and women as unwilling victims. This expectation of women’s sexual passivity may affect their ability to decline sex which they feel will be unpleasurable, unfulfilling or simply unwanted [15,18]. In addition, the limited research on how contraception affects sexual enjoyment and functioning, especially for women, is an indication of how gender beliefs about women’s role in sexual relationships (and their experience of pleasure) has been left out of sexual health discourse, even though research indicates that the way contraception makes sex feel is linked to its uptake and continued use [19,20].

There is also a growing body of work on how sexuality has been marginalised within development programmes, and how this limits the effectiveness of development efforts [21]. Particular calls have been made for a greater recognition of the role of sex and sexuality in HIV interventions [14,22].

### Re-thinking public health approaches

Despite the backdrop of dominant sex-negative public health practice, anecdotal evidence from a wide range of countries suggests that safer sex, including condom use, can be eroticised and made pleasurable [2,17,23]. Indeed, researchers and sexual health practitioners working within a wide range of contexts and cultures have been calling for a more pleasure-focused approach to sexual health, in part so that audiences will be more receptive to safer sex messages and recognise them as relevant to their own sex lives [e.g., [17,24-29]].

The World Health Organization [30] and the World Association for Sexual Health [5] now recognise sexual pleasure as a key component of sexual health.

And a recent qualitative study with young women in Zimbabwe posed the question:

“Shouldn’t public health practitioners be programming for pleasure, as it is primarily within these contexts of pleasure, rather than of danger, that many young women are probably at greater risk of picking up infection?” [31].

In terms of experimental studies, there have been calls for a more pleasure-focused approach from researchers investigating promotion of new prevention technologies. In particular, this has emerged unprompted from intervention trials of vaginal microbicides [32-35] and acceptability trials of the female condom [36-38]. In both cases, male and female participants have reported increased sexual pleasure and no loss of sexual pleasure when using these technologies, leading researchers to conclude that this has increased acceptance and usage of safer-sex technologies. According to Pool et al. [34]:

“First, products that are to be used in sex should not be separated from sexual excitement and pleasure. Second, products should be promoted and used for positive reasons (sexual enjoyment) rather than negative ones (fear of infection)” [[34], p2058].

In addition, a relatively small number of intervention trials have been conducted to test the impact of pleasure inclusion in sexual health promotion. For example, a randomised controlled trial which tested the effect of an erotic film on condom promotion showed significant increases in positive perceptions of condoms [39]. And a meta-analysis of erotic safer sex interventions found 21 studies conducted using experimental or quasi-experimental designs, in which participants showed significant risk reduction compared to controls, including a decrease in risky behaviour [40]. Most of these studies, however, involved sample populations of young Caucasians in North America; none were conducted with populations in Africa or Asia; and only one was conducted in a setting with high HIV prevalence (Brazil). While these studies provide some evidence of the impact of erotic components on sexual health, they have limited generalisability, especially to resource-poor settings and areas with high incidence of STIs. Thus there is a need for more research on the effectiveness of pleasure components in sexual health interventions in high-risk contexts [2].

This research could benefit from some engagement with or learning from the sex/pleasure and erotic entertainment sector. Erotic media is often maligned for reinforcing negative, limiting and harmful gender norms or unrealistic expectations of sexual relationships or bodies [41,42]. However, it is ubiquitous – in every country in the world – and the limited research which is available suggests that pornographic media and entertainment reaches a wide audience, including young people [42]. Yet rigorous evidence of the effects of sexually explicit media on sexual behaviour are extremely limited and what does exist is highly equivocal [42,43]. Outside of high-income/industrialised countries, research in this area is extremely rare. One exception is a study of condom use influences among men in Bangladesh, which found that men’s attitudes to condoms were strongly influenced by the lack of depiction of condoms in ‘Western’ pornography [44]. While one reaction to this finding could be to condemn or censor pornography – which is likely to be futile – it may be more useful to see it as a lesson for public health professionals to address the lack of depictions of condom use in pornographic media. However, such a positive approach is rare and the potential for delivering positive health messages through pornographic media is relatively unexplored [42,45].

It could be argued that, although erotic use of condoms and other forms of safer sex are infrequent in pornographic media, those that do exist could be among the only existing depictions of erotic safer-sex. Rarely in traditional sexuality education sessions would participants be shown how to put a condom on a real penis or (in the case of female condoms) in a woman’s vagina, especially in a a romantic, erotic or intimate manner. Yet this is what they may be expected to do once they leave the class. By contrast, skills building in most other areas, such as health provision, childcare and cooking, is usually built on observation and practice.

In summary, there is increasing evidence that sexual pleasure plays an important role in and could be a powerful tool in promoting sexual health. This is based on findings of research into safer-sex technologies such as microbicides, anecdotal and qualitative research and the few controlled trials on the subject. In addition, there is some evidence of the role of erotic media on attitudes to condom use, despite the woefully limited research into positive effects of erotic media. Nevertheless, the public health sector and the sex/pleasure industry (including erotic media) remain worlds apart in terms of knowledge transfer or engagement.

### A case study of The Pleasure Project

The Pleasure Project (TPP) began in 2004 as a campaign to build the evidence for and attention to a more honest and realistic approach to sexual health, particularly in the context of global health and development. Its founder, Anne Philpott, worked in sexual health promotion and education, with public health agencies in a number of countries, and with a condom company, and found almost no mention of sexual pleasure or even sex within these environments. However, when promoting condoms during educational workshops and trainings, Philpott and her colleagues found that people were more receptive to messages about how it *feels* to use condoms, rather than messages about prohibitions on sexual behaviour. For example, when promoting the female condom in a variety of countries including India, Sri Lanka, Senegal, South Africa and Thailand, people were more engaged when the discussion centred on how it can feel to rub the outer ring on the clitoris, or the inner ring on the end of the penis, than when the message was solely about how it can prevent pregnancy and STIs [2].

TPP’s aim is to increase the number of safe sexual acts around the world, and for pleasure, desire and eroticism to become ‘quality markers’ for sexuality education and sexual health interventions. It aims to make sex safer by addressing one of the major reasons people have sex – the pursuit of pleasure – and shifting the focus from disease, fear and risk, to ‘good’ sex. The organisation’s approach is based on identifying the reasons pleasure is excluded from health interventions, and the reasons people do not practise safer sex (e.g. many studies have shown that people believe ‘condoms reduce pleasure’), and finding ways to counter these tendencies through innovative and provocative communications.

TPP works to change the way sexuality education and sexual health interventions are viewed by public health practitioners – from a focus on disease prevention and risk to pleasure and health. While it is not alone in promoting pleasure as a component of sexual health, it has been a prominent player since 2004. During this time, TPP has worked to influence both policy and practice through research, advocacy and skills building which incorporate positive motivations for sexual activity into safer sex and sexual health programming and research agendas. TPP communicates sexual health research and information using edgy and erotic language and images – techniques used largely in advertising and marketing – to make sexual health information more enticing. It also takes a ‘show, don’t tell’ approach, for example, by demonstrating the use of a safer sex method using erotic language and describing condoms as sex toys rather than medical devices. TPP’s research and communications tools provide real-world examples of how safer sex can be (and in many cases, already is) eroticised in practice, and its case studies and other research are used as the basis for participatory exercises at workshops and trainings with sex educators, counsellors, non-governmental organisations (NGOs), health care providers and programmers.

Moreover, self-censorship, pre-conceptions and discomfort with issues related to sex and sexuality among public health professionals are common barriers to innovation in health promotion. Therefore TPP provides training to help increase the abilities of reproductive and sexual health practitioners to address sex and pleasure with clients, and to incorporate pleasure into curricula and education.

In terms of research, TPP currently lacks the resources to undertake controlled trials or other similar investigations of pleasure approaches. However, the identification and compilation of anecdotal and qualitative evidence on erotic approaches to safer sex should be seen as an important step in building an evidence base for effective sexual health practice. While influencing the research agenda to ensure pleasure components are included in controlled trials or other rigorous investigations is important, evidence-based practice is meant to be a two-way exchange – from research to practice/policy, and from practice to research [46]. Qualitative research, therefore, helps to illuminate ways that pleasure and eroticism are already being incorporated into health promotion in real-world settings, which can be instrumental in informing research design.

### Communications strategy

TPP’s primary focus has been to facilitate skills transfer between the worlds of health research, practice and policy, and the world of sex/pleasure (e.g., erotic media, sexuality activism, sex toys, sex work, sex blogs etc.). This puts the organisation in a ‘compromising position’ – between vastly different audiences, which at times have been diametrically opposed. To communicate effectively with such a broad spectrum of audiences, it is vital to target communications in specific ways and to build credibility in the eyes of different stakeholders.

To communicate with researchers and public health stakeholders, TPP has published articles in professional journals, including *The Lancet, Reproductive Health Matters* and *Development*; spoken at the Royal Society of Medicine in London and annually at international conferences in many countries (including international and regional AIDS conferences from 2004 to 2009); and is researching a literature review on safer sex and pleasure. In the past six years, more than 1,500 practitioners/decision makers have been exposed to pleasure training techniques and concepts and many more at conferences and through the media. TPP has conducted workshops and trainings with a number of organisations internationally, including CARE Cambodia and Share-Net (the Netherlands Network on Sexual and Reproductive Health and AIDS); and facilitated skills-building sessions at major conferences including five international AIDS conferences. Participants have been people who are keen to learn how to deliver their sex education in a more appealing way, people who want to negotiate safer sex more effectively or people who are initially intrigued to see explicit discussion at formal conferences. In addition, requests for training, articles and workshops have increased, including those with available funding. And TPP receives on average three to four email queries each month, from people in many countries (largely community-based organisations in low-income countries) asking for training materials or advice on incorporating sex-positive techniques into their work.

TPP conducts and teaches others to perform sexy condom ‘demonstrations’, which involve presenting a condom as a sex toy, and maintains a website and Blog [47]. The organisation has self-published two editions of the *Global Mapping of Pleasure*, a collection of 45 case studies of organisations and people in more than 15 countries who eroticise safer sex. The *Global Mapping* provides best-practice examples to other organisations looking for practical ideas for integrating a pleasure approach into their own work.

TPP communicates with the mass media and the general public by generating media attention, with articles about and interviews with TPP appearing, for example, in the *Washington Post, The Guardian, The Times, Sydney Morning Herald* and *Cosmo*. TPP has also been interviewed for radio and television programmes in Canada, Colombia, the UK, Spain and New Zealand.

Finally, one of the key audiences TPP aims to influence is the sex/pleasure world. TPP has worked with pornographic film directors and actors in the United Kingdom (UK) to help them integrate male and female condoms into their films as a part of the sexual storyline and sex play, rather than as ‘necessary evils’. And it has partnered with a British sex toy company, which donated a percentage of earnings from a range of products to TPP. In addition, the organisation has written articles and been interviewed for erotic magazines, including recent articles in *Playboy* (Philippines), and past articles in *Forum*. The UK’s Sexual Freedom Coalition nominated TPP for an Erotic Oscar in 2006. The TPP website offers sexy tips, which are useful to those in the sex industry as well as to the general public and sex educators.

### Challenges

Overall, the response to TPP’s work has been positive and inspiring. One of the big challenges, however, comes from the preconceptions of different stakeholders, which limits the organisation’s ability to secure funding or establish partnerships with stakeholders, despite shared goals. In the public health, NGO and academic context, there are a number of entrenched ideas that hinder efforts to promote the pleasure approach. For example, that:

• all erotic images and media are harmful to women;

• talking about pleasure means talking only about men’s pleasure;

• discussing sexual pleasure and health could lead to creating new (harmful) norms, such as that orgasm is essential, or it could lead to a narrow definition of pleasure;

• poor people (and especially poor women) are not interested in sex;

• women, in particular, are generally victims when it comes to sex, and rarely agents of their own sexuality and sex lives;

• we are imposing ‘western’ concepts of sex/pleasure/erotics on other cultures;

• sexy images and marketing are not ‘serious’ or worthy of study or consideration in public health;

• the lack of experimental research, particularly randomised control trials, on pleasure and safer sex in different countries and contexts means that it is not a subject worthy of policy attention.

While some stakeholders in the public health world view TPP as the ‘porn people’, those in the ‘pleasure’ world have sometimes referred to the organisation as the ‘condom pushers’. In addition to these narrow perceptions, some funders have shied away from supporting TPP. For example, a programmer in one organisation suggested that the NGO’s directors would not condone working with or funding an organisation associated with pornography. The lack of core and long-term funding remains a challenge and is exacerbated by the UK’s Charity Commission rejecting TPP’s application for charitable status in 2005, in part with the explanation that pleasure is not a charitable objective. However, this highlighted one of the key challenges TPP faces: the need to articulate that its ultimate objective is better health, and pleasure is the mode or tool used to promote health, rather than pleasure being an end in itself.

Another challenge in communicating about the potential of pleasure in sexual health promotion is the lack of existing indicators for measuring constructs of sexual pleasure [48]. While this is not unique to work on sexual health – many psychological constructs are difficult to operationalise in the form of valid and reliable indicators [e.g., [49]] – the culturally-defined and diverse nature of sexual pleasure poses significant challenges. This leads, in turn, to difficulties with establishing the efficacy of pleasure-oriented or positive approaches to sexual health, and thus with mobilising funding and support.

There is a risk that the paucity of existing evidence on pleasure approaches from controlled trials may lead to a belief among some donors, policymakers or practitioners that, because the approach is unproven, it is ineffective. This is a fundamental tension within the evidence-based discourse, both within medicine and social intervention – without rigorous evidence it can be difficult to mobilise investment [50]. Yet some of the strongest proponents of evidence-based practice argue that limited published evidence is an indicator of the need to investigate further [e.g., [51]], a concept which TPP embraces in pushing for more research to test pleasure and erotic components in prevention interventions.

### Finding gender- and culture-sensitive erotic images

Another challenge is finding images for communications materials which appeal to both the pleasure and health worlds, will not be viewed as discriminatory, and will be seen as ‘sexy’ by a diverse global audience. What is sexy and erotic, as well as pleasurable, is, of course, highly varied across genders, cultures and individuals, which make efforts to use eroticism to promote health difficult. Without funding to commission photographs for publications and the TPP website, the organisation worked with a professional graphic designer, who used fee-free 1950s pornographic cartoon images from Latin America to design the website. Still shots of scenes from pornographic films to which TPP provided consultancy have also been used, however these are largely images representing white, American/European women and men and popular ‘western’ beauty norms.

More recently, TPP commissioned photographs to be taken in India, under the supervision of an Indian photographer and Indian film actress, who was the model for the photos. The aim was to make the images sexy and eye-catching to a wide audience, and more culturally representative.

Focus groups and interviews were conducted with (mostly female) development professionals working in Europe and India to assess their reactions to the images (with funding from The Institute of Development Studies Sexuality and Development Programme, at the University of Sussex, UK). Surprisingly, the professionals working in Europe (mostly the UK) expressed nervousness about the use of explicit images of an Indian woman, suggesting that it could be construed as racist. The Indian women and other women who were working in development in India, on the other hand, felt the images were not explicit enough. Another reaction was that the images objectify women and therefore are harmful or negative; however, during the same focus group, a development professional from India suggested that, sometimes, people find objectification sexy.

Finding representations of ‘sexiness’ which can be used to promote health among a diverse audience remains problematic, and TPP has found the old adage true that ‘you can’t please all the people all the time’. This is particularly so with regard to people who oppose pornography in all its forms. However, the challenge of trying to appeal to diverse audiences has also pushed the organisation to innovate and led to the creation of unique tools and methods. For example, being invited to present at a meeting of the UK Sexual Freedom Coalition – alongside a stripper and others involved in sexual entertainment – led to the development of the ‘sexy condom demo’, whereby a TPP trainer presents the female condom as a sex toy rather than a medical device, highlighting the features which can provide pleasure. This method has subsequently been used in front of audiences of health professionals, such as at the Royal Society of Medicine and international AIDS conferences, thus transferring knowledge from one ’world’ to another.

### Measuring impact

TPP is run largely by unpaid volunteers, therefore determining its impact is crucial to the volunteers’ motivation, and the organisation’s sustainability and future prospects for funding. While there is a lack of resources and capacity to systematically measure impact, there are a number of outcome indicators which help illustrate potential change. These are proxy indicators of general impact; it has not been possible to assess direct policy impact. TPP has an ultimate goal of becoming redundant, as discussion of pleasure becomes a necessary indicator of quality sex education. The organisation is part of a growing pleasure/sex-positive movement in public health, which can be demonstrated through: the amount of media coverage and online references to relevant issues and to the organisation itself; the number of journal publications (by and about TPP, which reference its research, and on the subject of pleasure, safer sex and public health generally); presence of relevant issues at international conferences; and invitations (and funding) to facilitate trainings and sessions at conferences and other events.

Since 2004 there has been a noticeable increase in the number of abstracts and presentations featuring the combined concepts of pleasure and safer sex at public health conferences, particularly international AIDS conferences. For example, a simple search of the International AIDS Society’s conference proceedings database found that the number of presentations including the combined search terms ‘pleasure or pleasurable’ and ‘safer or safe’ increased from zero in 2006 to 11 in 2008; the number of accepted abstracts increased from 15 to 20; and the number of rapporteur reports increased from one in 2006, to three in 2008 (search performed via http://library.iasociety.org/GlobalSearch.aspx, on 2 November 2010). Anecdotally, when TPP hosted a satellite session at the 2004 International AIDS Conference in Bangkok, a number of colleagues working in public health warned TPP presenters they could get arrested for showing erotic films. Conversely, at conferences in 2006, 2008 and 2010, a number of attendees queried when and where TPP would show the films, while dire warnings from colleagues ceased. In addition, at the 2010 International AIDS Conference in Vienna, Austria, The Pleasure Project was invited to contribute to the final rapporteur summary session for Track D: Social and Behavioural Sciences, which synthesised presentations, critical issues and important results presented throughout the conference [52].

In the past six years, more than 1,500 practitioners/decision makers have been exposed to pleasure training techniques and concepts and many more at conferences and through the media. TPP receives more and more requests for training, articles and workshops, which have come with far more offers of funding. In addition, it receives, on average, three to four email queries each month for training materials and advice on incorporating pleasure into sexual health programming. These come from around the world, but usually from community-based organisations in low-income countries.

Since 2004, TPP has conducted monthly online searches using Google and the keywords ‘pleasure’ and ‘safer sex’. From 2004–2008, at least eight of the top ten entries could be linked back to TPP, including our publications, conference appearances and press reports. In 2009, however, TPP appears in six out of the top ten entries, while other organisations and individuals come up more frequently under ‘pleasure’ and ‘safer sex’. While Google listings are not systematic and can be random, the indication that others are doing pleasure work suggests a growing pleasure movement, and many of those search results have a connection with TPP. However, it is difficult to track direct influence.

### Quantitative indicators

Over a 14-month period (August 2008–September 2009), TPP’s website (http://www.thepleasureproject.org) had: more than 40,000 unique visitors; an average of 4,400 unique visitors each month, with spikes after conference appearances; nearly 150,000 page views; and visitors from 25 countries. The most popular page was the homepage (21%), and second most frequently requested page was Sexy Tips (11.8%). The most commonly downloaded file was the *Global Mapping of Pleasure*, which has been accessed 17,000 times since it was launched in August 2008.

### Surveys and testimonials

A general online survey conducted by TPP in April 2009 received 17 responses from a range of countries. Most respondents had heard about TPP at conferences or from colleagues. The most common responses when asked about perceived impact of TPP’s work were:

1. Respondents were more comfortable talking about role of pleasure in sexual health/safe sex.

2. Respondents were more knowledgeable about pleasure and safer sex/sexual health.

3. Respondents were better able to articulate issues related to pleasure and safer sex.

4. Raised awareness of pleasure/safer sex in the public health/HIV prevention sectors.

Comments included:

“The Pleasure Project and its principles support workers and give them more confidence in delivering positive safer sex messages. …” (Health promoter, UK)

“I saw [TPP founder] Anne Philpott’s presentation at the 2004 HIV conference in Bangkok while I was giving HIV education and prevention instruction in an immigration detention centre (with detainees and immigration police officers). I was already trying out a variety of activities to break down negative associations with condoms … Her presentation … had a lasting effect and … helped me seriously address some of my own negative associations with condoms. I really needed to do this as a young gay western man working in and around Thailand.” (Public health trainer working in Thailand)

In a workshop evaluation in April 2009, TPP received 10 responses. When asked to assess the usefulness of the workshop, respondents said it enhanced their abilities to:

• understand sex-positive approaches;

• explain to others the role of pleasure in sex education and sexual health programming;

• talk about sex and pleasure in the context of health and HIV prevention; and

• develop or run sexual health/HIV prevention programmes.

Catherine Montgomery, a researcher involved with microbicides trials at the London School of Hygiene and Tropical Medicine commented:

“I think the value of the Pleasure Project for us rests … on how we write up and present this data. The visibility of The Pleasure Project at international conferences is particularly important in creating an environment where it is not only legitimate but actually ‘in vogue’ to discuss disease prevention in terms of sexual pleasure.” (personal communication, A. Philpott)

Finally, at the ICAAP8 conference in Sri Lanka in 2007, a Catholic nun approached the TPP workshop facilitator, asking to see safer-sex erotic film clips. She said this “was her opportunity to really learn and see how safe sex is performed … an opportunity we are denied through any other form” and that this would be extremely useful in her role counselling for marriages.

## Conclusions

TPP has received a much warmer reception than its founders expected when the organisation was launched in 2004. There is increasing demand for its services, for example, through email requests for educational materials, and invitations to deliver trainings and workshops that are fully funded. Media attention has been substantial, especially considering that TPP is a small activist group operating solely through volunteers and with minimal funding. The organisation’s work has been widely quoted and there is enthusiasm from a broad range of audiences for its approach. However, policy change is far more difficult to measure than acceptance, and only proxy or distal indicators can be used to measure success. TPP’s ultimate success would be more people having ‘better’ and safer sex around the world, but this is also very difficult to measure and more work needs to be done to operationalise pleasure-oriented constructs.

TPP faces a number of challenges. For example, in trying to engage fully with the public health world and charitable sector, it is vital to ensure the organisation’s intentions are communicated clearly – that its ultimate objective is better health, and pleasure is the mode or tool used to promote health. Other challenges include mobilising funding and support despite limited existing rigorous research on pleasure and safer sex; discomfort among public health practitioners and researchers with concepts of pleasure and sex; and rejection of erotic media as a potential tool for prevention on the grounds that it is always harmful.

Despite the challenges, TPP’s experiences over the past six years can provide useful lessons for public health organisations and practitioners looking for new ways to communicate about health and prevention. In particular, the organisation has learned how to grab attention and deliver safer-sex messages and techniques to diverse audiences, and how to open up new avenues for public health campaigning, potentially (and hopefully) as a route to increasing safer sexual behaviour in different cultures and contexts.

In addition, by combining all levels of evidence – from the rigorous experimental to the anecdotal – with experiential knowledge from the sex industry and our own work as safer sex proponents, TPP hopes to continue providing a medium for skills transfer, thereby continuing to bridge the health–pleasure divide.

## Competing interests

This article critically reflects on a research project in which the authors have been involved.
